# Manipulating extracellular tumour pH: an effective target for cancer therapy

**DOI:** 10.1039/c8ra02095g

**Published:** 2018-06-19

**Authors:** Guanyu Hao, Zhi Ping Xu, Li Li

**Affiliations:** Australian Institute for Bioengineering and Nanotechnology (AIBN), The University of Queensland Brisbane Queensland Australia 4072 l.li2@uq.edu.au gordonxu@uq.edu.au

## Abstract

The pH in tumour cells and the tumour microenvironment has played important roles in cancer development and treatment. It was thought that both the extracellular and intracellular pH values in tumours are acidic and lower than in normal cells. However, recent progress in the measurement of pH in tumour tissue has disclosed that the intracellular pH (pH_i_) of cancer cells is neutral or even mildly alkaline compared to normal tissue cells. This review article has summarized the recent advancement in the measurement pH_i_ and extracellular pH (pH_e_) in cancer cells, and the effect of pH_i_ and pH_e_ on proliferation, migration and biological functions of cancer cells. This paper has also elaborated recent treatment strategies to manipulate pH_i_ and pH_e_ for cancer treatment. Based on the recent progress in pH_i_ and pH_e_ manipulation in cancer treatment, we have proposed potential nanoparticle-based strategies to manipulate pH_i_ and pH_e_ to effectively treat cancer.

## Abstract 2

The pH in tumour cells and the tumour microenvironment has played important roles in cancer development and treatment.

## Graphical abstract

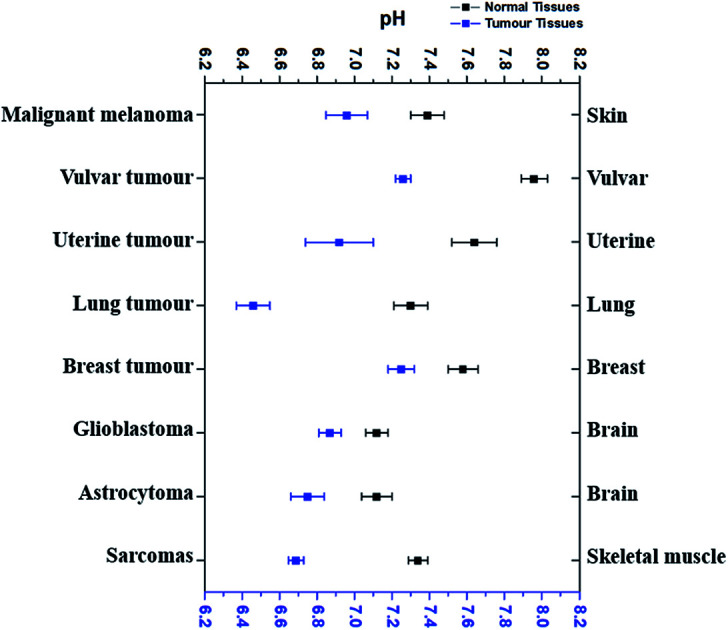

## 1. Introduction

Cancer is one of the most severe diseases in the world. According to statistics, in total 8.8 million people died from cancer in 2015, accounting for 17% of the total deaths.^1,2^ Researchers have made great efforts to understand the pathogenesis and properties of cancer in order to develop effective treatments for clinic application. As known, extracellular and intracellular pHs in tissues affect the function of the cells and play an important role in cancer development and treatment. As reported, the extracellular pH (pH_e_) affects the proliferation of human T cells and the expression of the interleukin-2 receptor.^3^

It is widely accepted that the pH_e_ of cancer cells is more acidic than normal cells.^4–6^ Generally, pH_e_ values of the normal tissues (brain tissues, subcutaneous tissues, *etc.*) are in the rage of 7.2–7.5. However, pH_e_ of tumour cells is mildly acidic in the range of 6.4–7.0. Since Warburg *et al.* first reported the abnormal anaerobic glycolysis in tumour cells, they measured the glucose and lactic acid in tumor veins and found more lactic acid and less glucose on the tumour tissue than on the normal tissue due to the fermentation process in the tumour side, which may affect pH_e_ and pH_i_ in tumour.^7^ Consequently, it has been assumed that pH_e_ and pH_i_ in cancer cells should be more acidic than those in normal cells during 1930s to 1980s.^7,8^

With the progress on sensing technologies, several techniques have been developed to measure pH_i_ and pH_e_ in cancer cells including pH-sensitive nuclear magnetic resonance spectroscopy (MRS), positron emission tomography (PET) radiotracers, magnetic resonance imaging (MRI) and optical imaging (Optics).^9^ It has been found that pH_i_ in cancer cells is actually mildly alkaline or near neutral, similar to normal cells.^10,11^ These new findings subvert the traditional assumption that the pH_i_ in cancer cells is more acidic than normal cells. More extracellular acidity and more intracellular alkalinity means a smaller ratio of pH_e_/pH_i_.

Subsequently, researchers have investigated the mechanisms of pH controls in cancer cells and microenvironments. Numerous membrane transporters across tumour cells have been found for pH hemeostasis in cancer cells, and further been used to manipulate pH_e_ and pH_i_.^2,12–17^ These novel strategies have been developed to control the pH_e_/pH_i_ ratio in cancer microenvironments and cells to induce apoptosis of cancer cells, improving the treatment efficiency.^18^

In this review, we have summarized the recent progress on the studies of pH_e_ and pH_i_ in tumour tissues and their corresponding normal tissues. Then, we have further outlined the mechanisms of pH_e_/pH_i_ maintenance in cancer cells and the developed therapeutics to manipulate the pH_e_/pH_i_ in cancer tissues. In the outlook, the potentials of new strategies using state-of-art nanotechnology to manipulate the pH_e_/pH_i_ in cancer tissues have been proposed for cancer treatment.

## 2. pH_e_/pH_i_ in tumour tissues *versus* normal tissues

### 2.1. Technologies for *in vivo* pH measurement and their accuracies

Several approaches for the measurement of pH_e_ and pH_i_ in tumour have been developed including pH-sensitive electrodes (POT), chemical exchange saturation transfer MRI (CEST-MRI), MRS, PET, MRI, and Optics.^4,6,19–33^Table 1 summarized some basic information of four major technologies for *in vivo* pH measurement.

**Table 1:** The technologies for *in vivo* pH measurement

Technology	First use	Accuracy (pH unit)	Mechanism	Major Advantage	Reference
POT	1950s	±0.1–0.2 pH	Use of pH-sensitive electrodes with tip diameters ranging from 0.5 pm to 2 mm	Electrodes can be directly controlled by hand and the results can be easily read	4 and 6
PET	1970s	±0.08 pH	Based on the presence of pH-dependent biologically active molecule	High sensitivity (nM–pM level detected)	23 and 24
MRS	1980s	±0.06 pH	Based on the pH-dependent chemical shift of the resonance frequency	Real-time observation of multiple metabolites	25 and 26
MRI	1990s	±0.1 pH	Based on the pH-dependent relaxation agent, hyperpolarized ^13^C-labelled agent, and/or proton-electron double resonance imaging	Visible, concentration-independent	19–22 and 27–29
Optics	2000s	±1.5% (±0.1 pH)	Based on the specificity of fluorescence probes and pH sensitivity of their emission lifetime	Non-invasive, Independent to the concentration of agent and intensity of the excitation light	31 and 32
CEST-MRI	2000s	±0.01 pH	Based on the agents that are capable of exchanging protons with the surrounding water molecules, lead to the continuous buildup of magnetic saturation of water, resulting in extremely enhanced sensitivity	Very low concentration extremely high sensitivity	21 and 33

Although several novel MRI and optical imaging agents (probes) have been developed and applied for *in vivo* pH measurement, there is not adequate data of the pH values measured by the same MRI or Optics method for comparable analysis. Thus, for the consistency of the comparison, the pH measured by POT or MRS were collected and compared in Section 2.2 and 2.3.

### 2.2. Extracellular pH (pH_e_)

**Fig. 1:**
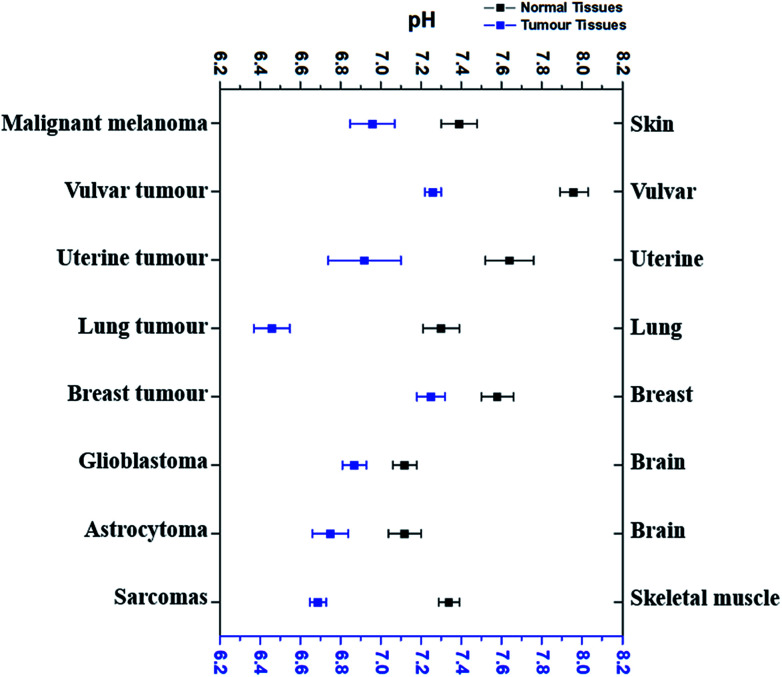
The comparison of average extracellular pH values of different tumours with normal tissues. Blue dots refer to the average extracellular pH of some cancer tissues, while black dots the average extracellular pH of corresponding normal tissues. All dots (average pH_e_ ± SEM) referred to the average extracellular pH of a specific kind of cancer or normal tissues listed. Data were taken from several different sources (ref. 4, 6 and 34–39), which were given in the text correspondingly.

According to the literature reports, pH_e_ of eight types of tumour tissues and the corresponding normal tissues has been summarized in Fig. 1. These data were selected based on the measurements using pH-sensitive electrodes.^4,6,34–39^

As shown in Fig. 1, pH_e_ of cancer cells is 0.3–0.7 pH unit lower than that of corresponding normal cells. For example, malignant melanoma tissues have an average pH_e_ of 6.96 while the average pH_e_ in normal skin cells is 7.39,^4^ which is 0.43 difference. The average pH_e_ in vulvar tumours is 7.26, 0.7 pH unit less than in normal vulvar tissues (with an average pH_e_ of 7.96).^6^ Uterine tumour tissues also have a lower average pH_e_ (6.92) than normal uterus, whose average pH_e_ is 7.64.^6^ Although the average pH_e_ of two kinds of brain tumours is slightly different, they are both more acidic than normal brain tissues.^38^ Similar results have also been observed in other tissues, such as lung,^34^ breast,^39^ and skeletal muscle.^35–37^ Thus, it is very clear that most cancer cells usually have a more acidic pH_e_ than their corresponding normal cells, and the differences vary from 0.3–0.7.

**Fig. 2:**
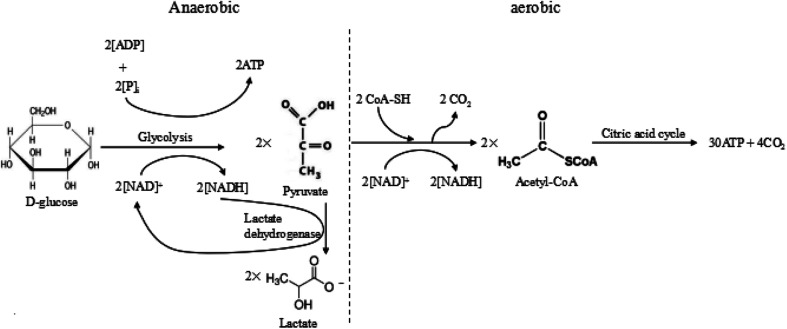
The anaerobic and aerobic pathways of glycolysis.

Warburg *et al.* proposed that tumour cells used glycolysis rather than oxidative phosphorylation to acquire energy, even in the presence of oxygen.^7^ Excess anaerobic glycolysis has been considered as the major reason for the extracellular acidity of tumour tissues.^10,40^ For most animal cells, there are two different pathways for glucose metabolism, *i.e.* aerobic and anaerobic glycolysis. The detailed processes of glucose metabolism in the cells have been briefly outlined in Fig. 2. There are two possible pathways for glucose metabolism in the cells: aerobic and anaerobic pathway. Generally, one glucose molecule is metabolized to two pyruvate molecules, producing two ATP molecules as the energy. In the aerobic pathway, two pyruvate molecules react with CoA-SH and form acetyl-CoA by releasing CO_2_. Subsequently, the produced acetyl-CoA undergoes the citric acid cycle, finally degrading into CO_2_ and producing 30 ATP molecules. In the anaerobic process, two pyruvate molecules transfer into two lactate molecules with the assistance of lactate dehydrogenase, but this transfer only produce 2 ATP. The overall reactions of these two ways are briefly expressed as follows:C_6_H_12_O_6_ (d-glucose) → 6CO_2_ + 6H_2_O + 38ATP (aerobic)C_6_H_12_O_6_ (d-glucose) → 2C_3_H_6_O_3_ (lactate) + 2ATP (anaerobic)

In the normal cells, most glucose is fully metabolized to produce carbon dioxide, water and the energy *via* the aerobic pathway. However, in the tumour cells, the glucose is mostly metabolized through the anaerobic pathway, which produces a large amount of lactate and releases limited energy due to a high level of pyruvate and hypoxia in the tumour environment. During the process, the tumour growth requires a large amount of energy compared to the normal tissue, which produces more CO_2_ and lactic ions in tumour. The produced CO_2_ was excreted extracellularly, resulting in the acidic condition in the tumour microenvironment, *i.e.* 0.3–0.7 pH units lower than the average pH_e_ of normal tissues.

### 2.3. Intracellular pH_i_: acidic or not?

**Fig. 3:**
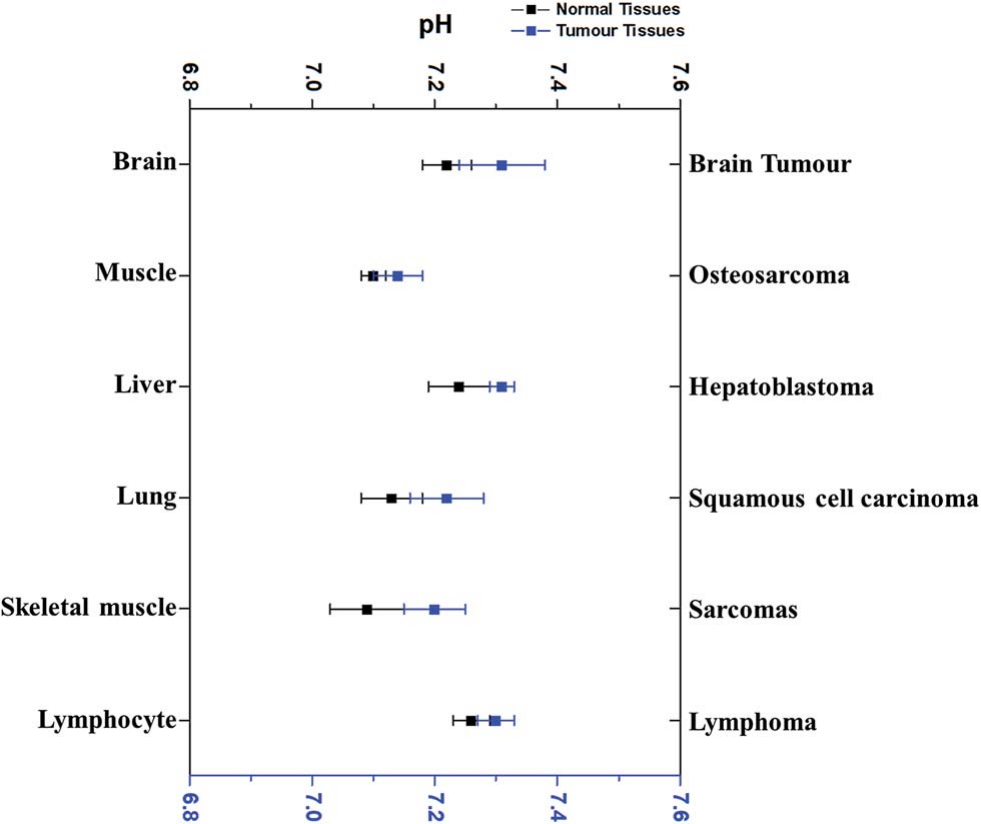
The comparison of the average intracellular pH values of different tumours with that in the corresponding normal cells. Blue dots refer to the average intracellular pH of tumour cells, while black dots represent the average intracellular pH of corresponding normal cells. All dots (average ± SEM) refer to the average intracellular pH of a specific kind of cancer or normal cells listed. Data were taken from different sources (ref. 11 and 41–45).

Interestingly, pH_i_ of cancer cells is not acidic, not as postulated previously. Since the 1980's, more research outcomes have demonstrated that pH_i_ of cancer cells is around neutral and even mildly alkaline.^10,11^Fig. 3 has displayed the pH_i_ of six kinds of tumour tissues and their corresponding normal tissues collected from MRS method.^11,41–45^

Very surprisingly, the average pH_i_ of these tumour cells is slightly higher than that in their corresponding normal cells, although the difference is less than 0.1 pH unit and not significant. For example, the average pH_i_ of brain tumours is 7.31, slightly higher than normal brain cells (7.24).^11,42,46^ Redmond *et al.* reported that the intracellular environment of osteosarcoma cells is also mildly more alkaline than in normal cells.^43^ Furthermore, this weak alkalinity of the intracellular environment in tumour cells has also been discovered in many other types of tumours, such as hepatoblastoma^11^ and squamous cell carcinoma.^44,45^ These evidences thus clearly indicate that pH_i_ of tumour cells is near neutron or even more alkaline. Thus, the discrepancy of pH_e_ and pH_i_ in tumour cells is much larger than in normal tissues.

### 2.4. How cancer cells maintain their unbalanced pH_e_/pH_i_ ratio?

For most cells, the maintenance of neutral (or mild alkaline) pH_i_ is achieved by transporting respiratory end-products (such as CO_2_ and lactate) across the cell membrane. When the extracellular concentration of acidic respiratory end-products is lower than intracellular, the excess CO_2_ can passively across the cell membrane by diffusion.^47^ However, in most cases, the CO_2_ and lactate generated from glucose metabolise is accumulated in extracellular tumour site due to low blood flow rate, resulting in development of acidic microenvironments in tumour.^48,49^ In this situation, the release of CO_2_ and lactate in microenvironments mainly relies on numerous special membrane proteins, such as carbonic anhydrase enzymes (CA2, CA9 and CA12). More relevant pH regulators are listed in Table 2 and discussed in Section 3. Overall, the maintenance of pH_e_ and pH_i_ is based on passive diffusion and active membrane transporters. Table 2 briefly summarizes some major pH regulators in tumours and their main functions, including anion exchangers (SLC4A1, SLC4A2, and SLC4A3), proton transporter vacuolar ATPase (V-ATPase), mono-carboxylate transporters (MCT1, MCT2, MCT3, and MCT4), sodium ion based chloride/bicarbonate exchanger (SLC4A8) and Na^+^/H^+^ exchanger 1 (SLC9A1).^50–59^

**Table 2:** The summary of some major pH regulators in cancer cells and their main functions in manipulating the ratio of extracellular pH and intracellular pH in tumour cells

Name	Description	Function	Reference
SLC4A1	Anion exchangers	Transport HCO_3_^−^ out of cancer cells	53 and 54
SLC4A2			
SLC4A3			
SLC4A7	Sodium bicarbonate cotransporters	Mediate the coupled movement of sodium and bicarbonate ions across the plasma membrane	55
SLC4A8	Sodium ion-based chloride/bicarbonate	Transport Cl^−^ out of tumour cells and simultaneously import HCO_3_^−^ into cancer cells powdered by Na^+^	56
SLC9A1	Na^+^/H^+^ exchanger 1	Transport intracellular produced H^+^ to the extracellular environment, and import Na^+^ at the same time	56
MCT1	Monocarboxylate transporters	Transport (both inside to outside and outside to inside) the products of glycolysis (such lactic acid and other monocarboxylates)	57 and 58
MCT2			
MCT3			
MCT4			
V-ATPase	Proton transporter vacuolar ATPase	A proton pump on the membrane of tumour cells, responsible for the stransportation of H^+^ between intracellular and extracellular plasma	59

In the last two decades, several complicated mechanisms have been revealed about how cancer cells maintain the alkaline pH_i_ and acidic pH_e_.^60–65^ Among them, the mechanism for the import of weak bases (*e.g.* bicarbonate) and the extrusion of weak acids (*e.g.* CO_2_, H_2_CO_3_, and lactate) with the assistance of proteins in tumour cell membrane has been clearly demonstrated.^66^ Apart from this, the intracellular protons have been pumped out of tumour cells in three different ways, including direct discharge from the cells, exchange with other extracellular cations (*e.g.* Na^+^), and extrusion by the vacuolar ATPase.^56,67^

## 3. The effect of pH_e_ and pH_i_ on tumour activity

As discussed above, the difference of pH_e_ and pH_i_ in tumour cells is much larger than in normal cells. The maintenance of pH_e_ and pH_i_ in the tumour mainly relies on some specific proton pumps and intracellular buffer systems.^10,68,69^ For instance, the balance of HCO_3_^−^/CO_3_^2−^ buffer system in tumour is administrated by carbonic anhydrase enzymes CA2, CA9 and CA12.^12,70,71^ Besides, the Na^+^/H^+^ buffer system is manipulated by Na^+^/H^+^ exchangers, such as SLC9A1.^72^ The regulation of pH_e_ and pH_i_ depends on the synergic effect of all of these pumps and buffer systems.

**Fig. 4:**
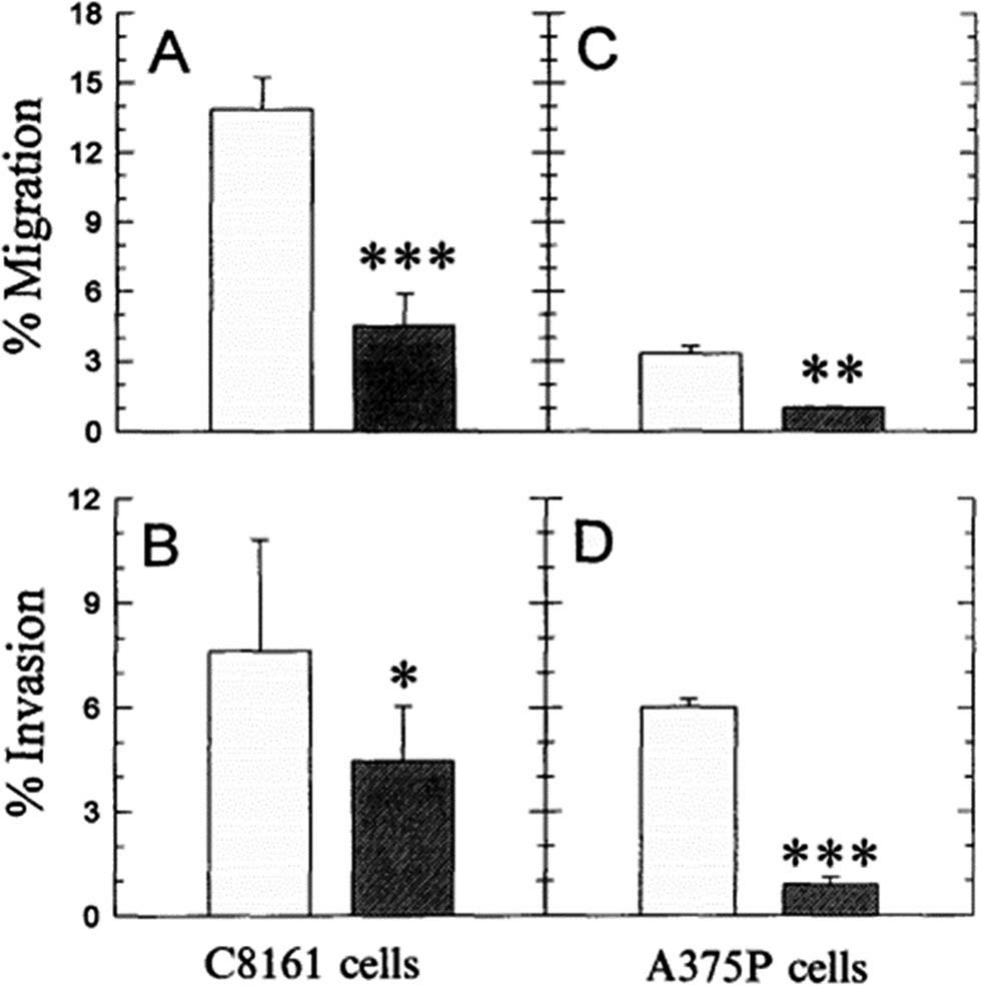
Comparison of the migration and invasion of C8161 and A375P cells treated in standard and acid media. The migration and invasion properties of cells treated in acidic medium (pH 6.8) were drawn in white bar, while black bar referred to the value of cells cultured in standard medium (pH 7.4). Data analysis was performed using Student's *t*-test: *P < 0.01; **P < 0.005; ***P < 0.001. This figure is adapted from ref. 73 with permission from Kluwer Academic Publishers.

**Fig. 5:**
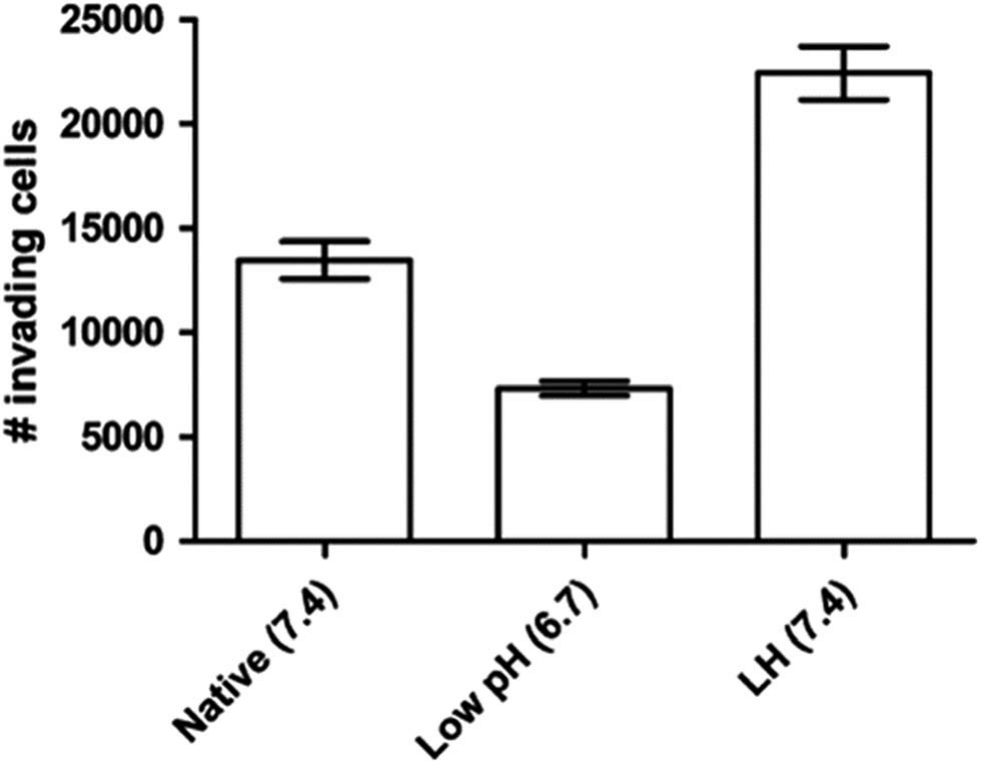
Invasion of different C8161 phenotypes. Representative invasion assay results for C8161 phenotypes assayed in their respective media. Native group meant the cells were incubated in normal medium. Low pH group represented the cells cultured in acidic medium (pH 6.7). LH group meant the cells were cultured in acidic medium for 1 month and then transferred into normal medium before the experiment. This figure is reproduced from ref. 74 with permission from Springer Netherlands.

It is known that even the little change of pH_e_/pH_i_ ratio may severely affect many biological and chemical processes in the cells, and eventually result in the proliferation and aggressiveness of cancer cells.^60^ For example, the incubation of melanoma in the acidic environment can significantly enhance its metastasis, aggressiveness and migratory activity *in vitro.*^73^ Martinez-Zaguilan reported that C8161 and A375P cells were cultured in acidic medium (pH 6.8) for 3 weeks and then transferred to the membrane invasion culture system (MICS) chambers.^73^ They found that C8161 cells and A375P cells treated in acidic medium have significantly enhanced migration and invasion, as shown in Fig. 4. Moellering *et al.* also reported that acidic-treated C8161 cells cultured in normal medium (pH 7.4) showed higher aggressiveness than those cultured in acidic environment (low pH group) and control (native group), as shown in Fig. 5.^74^ The C8161 cells cultured in lower pH medium (6.7) has shown the inhibition of the cell invasion, indicating less aggressiveness. These results have demonstrated that the regulation of pH_e_ and pH_i_ ratio in the tumour is highly important for metastasis, aggressiveness and migratory activity. Fine control of pH_e_ and pH_i_ in tumour may improve the cancer treatment.

**Fig. 6:**
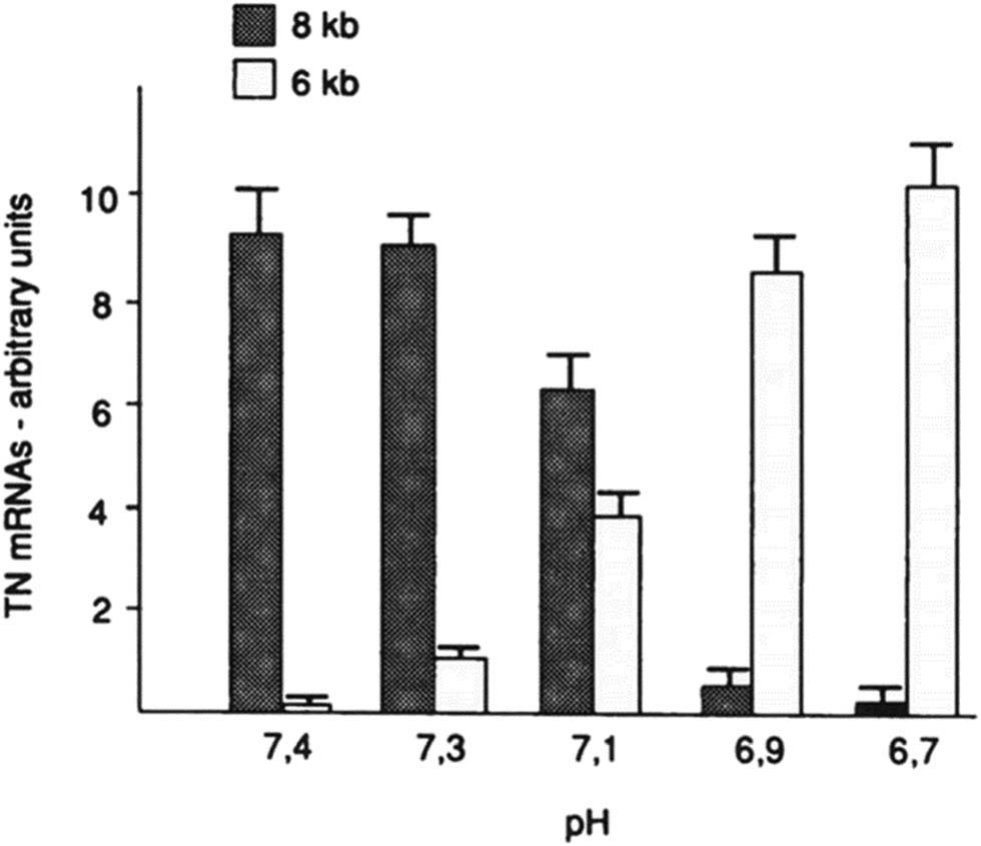
Effect of environmental pH on the RNA alternative slicing of TN-C mRNA isoforms in human skin fibroblasts. Skin fibroblasts were incubated in DMEM medium at different pH for 4 days, and the expressed TN-C mRNA amounts were derived from northern blot analyses and shown in arbitrary units. This figure is adapted from ref. 75 with permission from American Society for Biochemistry and Molecular Biology.

Furthermore, the slight change of pH_e_ and pH_i_ may also disorder the function of some proteins (such as tenascin and fibronectin), particularly in cancer cells.^75,76^ For example, mild change of environmental pH by 0.7 pH unit dramatically affected the RNA alternative slicing. The major expression of tenascin-C (TN-C) isoforms was 8 kb TN mRNA in human skin fibroblasts at pH 7.4, while 6 kb TN mRNA isoform was the majority of TN-C expression at pH 6.7 (see Fig. 6).

Tumour microenvironment triggers the tumour heterogeneity during the cancer development. It is well known that acidic condition and hypoxia are important characteristics in the tumour microenvironment. The homeostasis of pH_e_ and pH_i_ is very important for all kinds of cells. As discussed above, compared with normal cells, cancer cells have a more acidic pH_e_ and more alkaline pH_i_, suggesting that the pH homeostasis regulation of tumour tissues may be more complex and involve in more proteins and buffer systems. The pH environment may influence the growth and function of the cells in two main ways. On the one hand, the 0.1 alteration in the ratio of pH_e_/pH_i_ may affect many essential biochemical processes in the cell metabolism system, such as ATP synthesis, cell proliferation, aggressiveness, migration and diffusion, and the function of some membrane proteins.^60^ On the other hand, the tiny disturbance of pH_e_ may activate the mechanism of alternative splicing of constituents in extracellular matrix to produce isoform of tenascin and fibronectin, which specifically occur in cancer cells rather than in normal cells.^75,76^ Although the isoforms of these alternatively spliced proteins do not involve in the manipulation of tumour's pH_e_/pH_i_ ratio, they may provide binding sites for antigen-based cancer therapy.^18^

## 4. Strategies to manipulate the pH_e_/pH_i_ ratio

As discussed above, the small change in pH_e_/pH_i_ ratio of tumour cells may disturb many biological functions, including proliferation, aggressiveness, and migration. This relationship demonstrates that adjusting the pH_e_/pH_i_ ratio in the tumour tissues may halt cancer progress or even completely inhibit cancer growth. In recent years, several approaches have been developed to manipulate pH_e_/pH_i_ ratio for cancer treatment. These approaches can be classified as direct manipulation and indirect manipulation. Direct manipulation is to regulate the pH_e_/pH_i_ ratio of tumour cells by using acidic/alkaline drugs and indirect manipulation is based on operating the pH regulators of tumour cells.

### 4.1. Direct manipulation using small molecule drugs

The drugs for direct manipulation are mainly small molecular substances (such as bicarbonates). This approach is to directly increase pH_e_ of tumour tissues to the normal level (0.3–0.7 pH unit). It can be achieved by oral administration of alkaline agents or even by simple adjustment of diet habit.

The alkaline agents include sodium bicarbonate and trisodium citrate.^77^ In practice, it seems difficult to maintain the mildly alkaline microenvironment near tumour tissues *via* oral administration, as a high dose and continuous intake of the alkaline substrate is required. Based on the breast cancer study, White *et al.* investigated the exact daily dose of sodium bicarbonate needed for breast cancer treatment.^78^ The calculated daily dose for a normal human (with 70 kg weight) would be 31.75 g sodium carbonate or 32.5 g trisodium citrate.^79^ Another example is the Tris–base buffer to inhibit tumour progression and metastasis.^80^ The size of the pancreatic tumour in the mouse model was significantly decreased after 200 mM of Tris–buffer treatment. Based on their data, the daily dose for the mice can be calculated as 18.2 g of Tris–base buffer per kg, equivalents to 31 g of Tris–base intake per day for an adult (70 kg). Although it is possible for a cancer patient to intake more than 30 g alkaline agents (such as sodium carbonate or trisodium citrate) with daily drinking water, it would be more efficient to deliver alkaline agent to the tumour tissues rather than to the whole body. A recent non-randomized controlled study investigated the efficacy of local infusion of alkaline agent.^81^ Researchers found that there was a 6.4-fold difference of geometric mean of viable tumour residues (VTR) when the hepatocellular carcinoma patients were treated with transarterial chemoembolization (TACE) accompanied with or without locally infusing bicarbonate (LIB) into tumour (Table 3). Such a local administration may be a better strategy for anticancer therapy.

**Table 3:** The geometric means of viable tumour residues after different treatment of 57 patients with hepatocellular carcinoma. This table has been adapted from ref. 81 with permission from eLife Sciences Publications

	TACE (*n* = 27)	TACE + LIB (*n* = 30)	*P* value
Crude VTR	45.1% (30.3–67.0%)	7.1% (4.4–11.5%)	<0.0001
Multivariable adjusted VTR	45.6% (28.9–72.0%)	7.1% (4.6–10.9%)	<0.0001

The adjusted diet could be low in protein but high in potassium and/or magnesium.^82–84^ It has been proved that potassium can effectively neutralize mineral acidity and even mildly alkaline pH of urine *via* KHCO_3_ generation or glutamine sparing.^85^ The pH_i_ may be altered by a large change of the intake of potassium due to its fundamental physiologic and metabolic importance.^85^ Based on another big data analysis (based on more than 300 000 cases), the risk of suffering from pancreatic cancer decreased by 18% for each 100 mg increase of magnesium intake per day by men on the continuous scale.^86^ These results may provide a diet-based way to manipulate the pH environment *in vivo* and assist cancer treatment.

### 4.2. Indirect manipulation: proton pump inhibitors

The second alternative strategy to administrate the pH_e_/pH_i_ ratio is to inhibit the functional proton pumps. It is well known that the maintenance of high pH_e_/pH_i_ ratio in tumour tissues relies on many proton regulators (pumps) on the cell membrane. Most of these proton pumps on the tumour cell membrane have a few specific isoforms that do not exist on the normal cell surface. Thus these isoforms may provide some specific target sites for cancer therapy. Once these functional proton pumps are inhibited, the pH balancing system of tumour cells may be disordered and the pH_e_/pH_i_ ratio may increase. The abnormal proton transportation and change of the pH_e_/pH_i_ ratio may affect the behaviour of tumour cells. Recent research reports have demonstrated that the inhibition of proton regulators have suppressed the proliferation and promoted the programmed cell death in some tumour cell lines.^87–92^ For example, treatment with proton pump inhibitors led to the induction of apoptosis in many types of gastric cancer cells, which involves in the regulation of tumour pH.^89^ Besides, the inhibition of proton extrusion by Na^+^/H^+^ exchanger inhibitors^72^ or V-ATPase inhibitors^93^ may make cancer cells susceptible or vulnerable. Now a few proton pump inhibitor drugs have been used in the clinical stage. Table 4 lists some inhibitors and their target proton pumps.^90,94–102^ As seen in Table 4, the current inhibitor drugs mainly focus on two major pH regulators (V-ATPase and SLC9A1) and only one of these drugs, cariporide, has been successfully developed to phase III clinical trial.

**Table 4:** The summary of some inhibitors of major pH regulators (V-ATPase and SLC9A1) in cancer cells and their main functions in manipulating the intracellular pH in tumour cells and their current development stage

Inhibitors drugs	Identification site	Function & description	Reference
Omeprazole, esomeprazole	V-ATPase	Can be activated in the slightly acidic environment, and then inhibit V-ATPase *via* covalent interaction. Work on V-ATPase at high dose	94 and 95
Bafilomycin	V-ATPase	Commonly inhibits V-ATPase (not selective for tumour cells) with high cell toxicity	96 and 97
Diuretic amiloride	SLC9A1	Inhibits NHE-1 with unacceptable high concentration	98
EIPA (derivative of amiloride)	SLC9A1	200 times stronger than amiloride, has not used in clinical trial yet	98 and 99
Cariporide	SLC9A1	Decrease the intracellular pH of cancer cells. Has been developed to the third stage of clinical trial	90 and 100–102

Interestingly, decreasing pH_i_ may increase hyperthermia efficacy (over 42 °C) and the programming cell death response to TNF (tumour necrosis factor) induced by apoptosis ligand, also known as TRAIL.^96,103–105^ For example, both bafilomycine A1 (an inhibitor of V-ATPase) and EIPA (an inhibitor of the Na^+^/H^+^ exchanger) increased the thermo-sensitivity of the AsPC-1 tumours (grown in nude mice) by individually mildly decreasing pH_i_, and the thermo-sensitivity was markedly enhanced by the sharp decrease in pH_i_, resulting from the synergetic effect of the combination of these two therapies.^96^

**Fig. 7:**
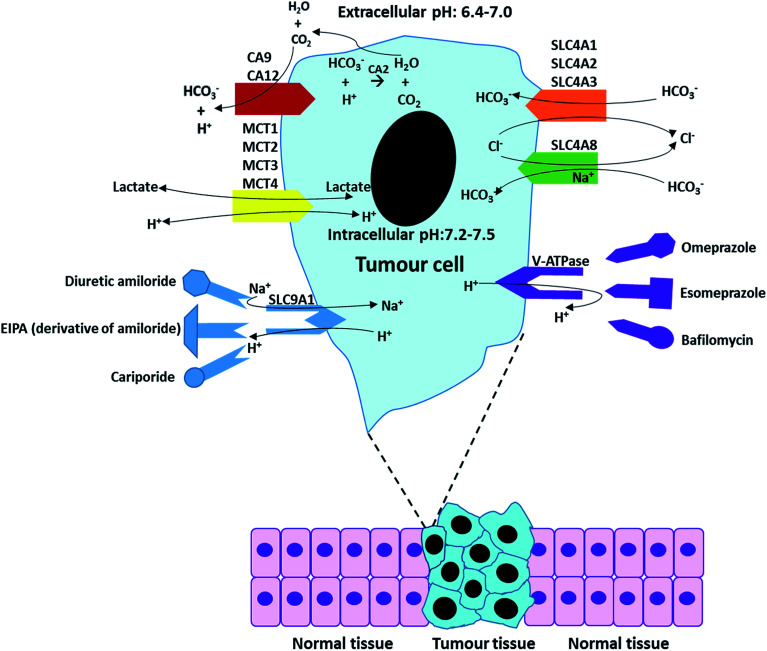
A summary of the regulation processes in a tumour cell and some “manipulators (inhibitors)”.

Fig. 7 outlines the functions of some specific pH regulators and relatively main inhibitors for tumour cells. The functions of these ion exchangers and proton pumps, and their main inhibitors have been described in Tables 3 and 4 The overall process of ion exchangers is the cellular intake of HCO_3_^−^ and cellular exhaust of CO_2_ and Cl^−^, which both lead to pH_e_ decrease and pH_i_ increase. Proton pumps (or more exactly Na^+^/H^+^ exchangers) directly exchange intracellular H^+^ with extracellular Na^+^.

To conclude, more targeting sites and relevant inhibitors need to be explored in order to more efficiently manipulate pH_e_/pH_i_ in tumour cells for potential and effective cancer therapy.

### 4.3. Alternative methods to manipulate the pH_e_/pH_i_ ratio

Several research reports have showed that the apoptosis of tumour cells can be boosted by the adequately large decrease of their pH_i_.^87–92^ One way to achieve the reduction of pH_i_ in tumour cells is to promote cancer glycolysis to the utmost extent by maximizing the glucose supplement. The extremely high rate of glycolysis may break the capacity of proton pumps in tumour cells, which means that tumour cells cannot timely transport acidic metabolites (such as H^+^, H_2_CO_3_, lactate *etc.*) outside and hence decreases pH_i_. For example, a very high glycolysis rate was observed in human melanoma cells (cultured in the medium containing high amount of glucose) when DNP, an uncoupling agent, was added.^106^

The programmed cell death can also be activated by the sharp reduction of pH_i_, finally leading to cell death.^102^ This may result from several different mechanisms, one of which is the reduction of glycolysis metabolism.^107^ For example, the enzymatic functions of hexokinase, one of the vital enzymes for the maintenance of the high level of glycolysis metabolism in tumours cells, was strongly inhibited (activity decreased from 82 ± 3.2% to 31.2 ± 5.7) with the sharp decrease (from 8 to 6 respectively) of pH_i_ in SNB-19 glioma cells.^108^

The tumour glycolysis can be promoted by inhibiting the production of mitochondrial ATP, which requires some specific inhibitors. *meta*-Iodobenzylguanidine is one of the inhibitors of mitochondrial complex 1, acting as proton extrusion inhibitors (or hyperglycemia) and then decreasing pH_i_ in cancer cells.^100,109–111^ However, this drug is normally used as a radioiodine therapy agent, and the dose used for radioiodine therapy is not high enough to perform a strong inhibition on proton transportation. Dinitrophenol (DNP), a new type of chemotherapeutic drugs, has also shown a remarkable enhancement in glycolysis with the increase of blood pressure at a low dose. It has been reported that mM-level DNP can inhibit the proliferation of cancer cells and lead to apoptosis in the human pulmonary adenocarcinoma Calu-6 cell line.^112^

Overall, even though there are some drugs (such as *meta*-iodobenzylguanidine and DNP) that have shown their ability to decrease pH_i_ by boosting the glycolysis rate in tumour cells, the hyperglycemia-reliable mechanism restricts the feasibility of this cancer therapy strategy.

## 5. Conclusions and future prospective

In this review, pH_e_ and pH_i_ in tumour cells have been summarized and the ways to manipulate cellular pH in cancer cells have been discussed. It is clear that tumour cells have a more acidic pH_e_ (0.3–0.7 lower) than normal cells, and pH_i_ in tumour cells is neutral or even more alkaline than that in normal cells. The abnormally high ratio of pH_e_/pH_i_ in tumour cells is due to the high rate of glycolysis in tumour cells, which produces numerous acidic products (such as H_2_CO_3_ and CO_2_). The maintenance of pH_e_/pH_i_ relies on several special proton pumps on tumour cell membranes, such as SLC9A1 and V-ATPase. Then the mechanisms of these proton pumps are discussed and two potential pH manipulating strategies are presented, including direct manipulation by delivering small molecule drugs and indirect manipulation sing proton pump inhibitors.

It has been demonstrated that treatment of cancers (halting its proliferation, aggressiveness and even inducing programmed cell death) is very possible by manipulating pH_e_/pH_i_ ratio in tumour. A future potential method is to combine 2 or 3 inhibitors so that pH_e_/pH_i_ can be well controlled, which may significantly enhance the efficacy of the cancer treatment.

The other future approach to manipulating the pH_e_/pH_i_ ratio for cancer treatment is to use functional nanoparticle delivery systems to efficiently transport the known inhibitors. Compared to small molecular inhibitors, nanoparticles could have more advantages. For example, nanoparticles can be accumulated around tumour tissues through enhanced permeability and retention effect (EPR effect).^113^ Of course, inhibitor-loaded nanoparticles can be further functionalized with target ligands, which may enhance the accumulation in the tumour tissues and manipulate the pH_e_/pH_i_ ratio.

Another potential way to direct pH manipulation can be achieved by target delivery of alkaline nanoparticles to the tumour tissues by virtue of EPR effect. Thus, accumulated alkaline nanoparticles neutralize the extracellular acids and efficiently increase pH_e_. Moreover, some alkaline nanoparticles can be modified as a carrier for delivering anticancer drugs to more efficiently treat cancers.^114,115^

## Conflicts of interest

There are no conflicts to declare.

## Supplementary Material
